# Hepatitis B vaccination status and Needle-stick and Sharps-related Injuries among medical school students in Nepal: a cross-sectional study

**DOI:** 10.1186/1756-0500-7-774

**Published:** 2014-11-03

**Authors:** Suraj Bhattarai, Smriti KC, Pranil MS Pradhan, Sami Lama, Suman Rijal

**Affiliations:** B.P. Koirala Institute of Health Sciences (BPKIHS), Dharan, Nepal; Ishan Children and Maternity Hospital, Kathmandu, Nepal; BPKIHS, HIV & SRHR Coalition, Lalitpur, Nepal; Influenza Surveillance Project, Department of Community Health Sciences, Patan Academy of Health Sciences, Lalitpur, Nepal; School of Nursing, BPKIHS, Dharan, Nepal; Internal Medicine, Tropical and Infectious Diseases Division, BPKIHS, Dharan, Nepal

**Keywords:** Hepatitis B vaccination, Needle-stick and Sharps-related Injuries, Universal precautions

## Abstract

**Background:**

Hepatitis B is a dreadful infectious disease and a major global health problem. Health-care workers including clinical students are more vulnerable to such infections and non-sterile occupational exposures as their daily activities are closely related to patient’s blood and body fluids.

**Methods:**

A descriptive cross sectional study was conducted at B.P. Koirala Institute of Health Sciences (BPKIHS), Dharan, Nepal from July till October 2012. All medical, dental and nursing students were surveyed for their Hepatitis B vaccination status and only those students in clinical rotations were surveyed for the prevalence and pattern of Needle-stick and Sharps-related Injuries (NSSIs) using a pre-tested, semi-structured, self-administered questionnaire. Descriptive and inferential statistics was used to analyze the data.

**Results:**

Majority (86.5%) of students were vaccinated against Hepatitis B of which 83.7% had completed full doses. Among non-vaccinated students, 43.2% reported the main reason for non-vaccination as lack of vaccination programs. Out of 210 respondents from clinical rotations, 90 students (42.8%) reported at least one injury. Among those injured, two students reported exposure to Human immunodeficiency virus (HIV) positive cases and four to Hepatitis B virus (HBV) positive cases. Most of the injuries (44%) occurred during Internal Medicine rotation and the most common sharp involved (56.3%) was Hypodermic needle. Most injuries (35.6%) occurred while manipulating needle into patients. Following exposure, only 11.4% took Post exposure prophylaxis and 19.54% went for a Post-exposure serology test.

**Conclusions:**

Needle-stick and Sharps-related Injuries occur frequently among health care workers including trainee students keeping them at high risk for acquiring dreadful infections like HBV, HCV and HIV. They need to be protected from unwarranted hazards by adopting routine Hepatitis B vaccination programs and by reinforcing education regarding universal precautions.

## Background

Hepatitis B is a dreadful infectious disease and a major global health problem. It can be asymptomatic, symptomatic or potentially fatal and can take the form of an acute to chronic liver disease. Nearly 2 billion people have been estimated to be infected with HBV worldwide and more than 240 million people have chronic liver infections. About 600,000 people die every year due to consequences of viral hepatitis B, either acute or chronic [1, 2].

Hepatitis B virus is spread by contact with infected blood or body fluids – from mother to child, child to child, parenteral routes, transfusion of blood or blood products and unprotected sexual intercourse. [2] HBV can also remain on any surface it comes into contact with for about a week for e.g. on table tops, blades, blood stains without losing its infectivity [1].

Hepatitis B is a vaccine preventable infection and the vaccine has been available in the world since 1982. The HBV vaccine is 95% effective in preventing infection and its chronic consequences. It is the first vaccine against a major human cancer i.e. Hepatocellular Carcinoma [1]. World Health Organization (WHO) has recommended vaccination for all infants, all children and adolescents younger than 18 years age and for all high-risk groups which include people with high-risk sexual behavior, partners and household contacts of infected ones, injecting drug users, people who frequently require blood or blood products, recipients of solid organ transplantation, people at occupational risks of hepatitis B virus infection, and travelers to the countries with high rates of hepatitis B infection [1].

In Nepal, about 315,000 people are estimated to be living with chronic hepatitis B infection [3]. However; the exact data of acute infections are unavailable in the national directory. Although National Immunization Program introduced Hepatitis B vaccination in 2003, an accurate immunization status of the population is also still unknown. So far, it is being administered as a component of Pentavalent injection (DPT-HepB-Hib) to the infants only [4]. The government doesn’t have any program to vaccinate at-risk population such as Health-Care Workers (HCWs). The health institutions have also failed to ensure protection of their employees and trainees.

In-service activities of a HCW are closely related to patient’s blood or body fluids. They are likely to sustain non-sterile exposures either in the form of needle-sticks, sharps-related injuries or splashes of body fluids to eyes and mucus membranes. If one sustains a needle stick and the source (patient) is an infected one, the risk of transmission of HIV, HBV and HCV per exposure is 0.3%, 37-62% and 1.8% per exposure respectively [5]. It is estimated that Sharps-related injuries cause about 66,000 HBV, 16000 HCV and 200–5000 HIV infections each year among HCWs globally [6].

The present study aims at exploring Hepatitis B vaccination coverage among medical, dental and nursing students of a Medical school in Nepal, the main reasons for non-vaccination and the association between vaccination status and socio-demographic variables. It also aims at studying the prevalence and pattern of NSSIs among trainee students.

## Methods

It was a cross-sectional study conducted in BP Koirala Institute of Health Sciences, Dharan, Nepal from July 2012 to October 2012. BPKIHS is a 700 bedded multispecialty, teaching and referral hospital located in Eastern Development Region of Nepal. Dharan is a major city of Eastern Nepal and represents the geographical transition between the long ranges of mountains in the North and the extensive plains of Terai belt in the South. The city has both cultural and socio-economic diversity. The local people rely on BPKIHS for their health needs. According to national surveys, the population of this region has relatively higher prevalence of infectious diseases like HBV, HCV and HIV as compared to the other parts of the country.

In BPKIHS, medical and dental students start hands-on training during internship rotations whereas nursing students start it right from the beginning of course. Our study was conducted among the students who represented the batches from 2006 to 2012.

### Study population and sample size

#### Hepatitis B vaccination status

All medical, dental and nursing students of BPKIHS ranging from Batch 2006 to Batch 2011 and willing to participate in the study were included in our study. There were overall six batches of medical and dental students (Batch 2006–2011), four batches of B. Sc. Nursing students (Batch 2008–2011) and three batches of Certificate Nursing students (Batch 2009–2011). The number of students enrolled each year in each batch of medical, dental, B.Sc. Nursing and Certificate Nursing faculty is 100, 40, 20 and 40 respectively. Those students who were absent during two-week period of data collection were excluded from the study.

### Prevalence of Needle-stick and Sharps-related Injuries (NSSIs)

All medical/dental interns of 2011/2012 academic session, who had completed 12 months of compulsory rotatory internship by August 2012, and all nursing students (both B.Sc. and Certificate Nursing students from first year through final year) were included. Medical and dental students start hands-on training usually during internship whereas nursing students do it from the beginning of their course. Interns and the nurses absent at the time of data collection were also excluded. The sample size was calculated on the basis of a similar study carried out in School of Population Health, University of Melbourne where overall prevalence of Needle-stick injury among Health-care workers was 63% [7]. We calculated the sample size of 226 at 95% confidence and 10% allowable error and considering 20% of non response, we calculated our final sample size to be 270.

Simple random sampling method was adopted while taking samples.

### Research instrument

A semi-structured questionnaire was developed including questions on hepatitis B vaccination status and NSSIs. The questionnaire was categorized into 4 sections: section 1- socio-demographic profile, section 2- Hepatitis B vaccination details, section 3- reasons for non-vaccination, section 4- NSSIs details. Only the interns and nursing students were requested to fill up section 4.

Sections 4 had different sub-headings: the prevalence of NSSIs, types of instruments involved in injury such as hypodermic needle, scalpel or a suturing needle; procedures involved such as drawing venous or arterial blood, suturing wounds, conducting labor, dental extraction; circumstances of injury such as while recapping used needle, manipulating needle back and forth into patient, administering intravenous or intramuscular medication, accidental injury by others, cleaning up and disposal; and causes of injury such as stress at work, understaffing, not following universal precaution, uncooperative patient.

The content validity of the research instruments was ensured after reviewing the literature and consultation with research guides, advisors and experts on the subject. Based on their opinions, modification was done accordingly.

The questionnaire was pretested by taking 10% of the sample. Questionnaires were distributed in the classrooms. A detailed briefing regarding questions and objectives of study was made prior to data collection.

### Definition of the variables

#### HBV vaccination status

Students are either vaccinated or not vaccinated. Those who received less than 3 doses of HBV vaccination by the time of data collection were categorized as partially vaccinated ones.

#### Needle-stick and Sharps-related Injury (NSSI)

An injury with a needle, scalpel blade, catheter stylet or other pointed object which was used for any invasive procedure with a patient and contaminated with blood or body fluids.

These two were considered as dependant variables whereas faculty, batch, age, sex and nationality of participants as independent variables. Faculty was divided into medical (MBBS), dental (BDS) and Nursing (both B.Sc. and Certificate). Batch varied from 2006 to 2011 including interns of 2011/2012 session. Majority of students in this institution were Nepalese followed by Indians.

### Data analysis

The coded data was entered in Microsoft EXCEL 2007 and transformed in SPSS (SPSS Inc. Released 2007, SPSS for Windows Version 16.0 Chicago, SPSS Inc.). Both descriptive statistics (frequency, median, mean, inter-quartile range, standard deviation and percentage) and inferential statistics were used to represent the data. Chi-square test was applied in bi-variate analysis for association among categorical variables; the probability of significance was set at 5%.

### Ethical consideration

Our research team obtained written permission from Institution Ethical Review Board (IERB) of BPKIHS to carry out the study. The objectives of study were explained to the participants and a written consent was obtained from each respondent prior to data collection. The participation was entirely voluntary and confidentiality of the responses was duly maintained.

## Results

Out of 930 students we approached to assess HBV vaccination status, 622 returned the answered questionnaires which gave the response rate of 66.9%. Among them, 20 responses were incomplete. Therefore, only 602 became valid for analysis. Likewise, the response rate for the details of Needle stick and sharps related injury was 73.3% (210 out of 270 questionnaires distributed).

### Hepatitis B vaccination status

#### Socio-demographic characteristics

Among 602 respondents, 55.1% were male and 44.9% were female. Majority of them (62.9%) were ≤22 yrs age and the mean age ± SD was 21.6 ± 2.215 years. More than half of the respondents were medical students (56.5%). Dental, BSc. Nursing and Certificate nursing students were 16%, 12.1% and 15.4% respectively (Table 1).

**Table 1:** **Socio-demographic characteristics of students in BPKIHS (n = 602)**

Description	Category	Frequency	Percentage
Faculty	MBBS	340	56.5
	BDS	96	16.0
	B.Sc. Nursing	73	12.1
	Certificate. Nursing	93	15.4
Batch	2006	44	7.3
	2007	42	7.0
	2008	103	17.1
	2009	136	22.6
	2010	130	21.6
	2011	147	24.4
Sex	Male	332	55.1
	Female	270	44.9
Age	≤22 yrs	379	63.0
	>22 yrs	223	37.0
Nationality	Nepali	544	90.4
	Indian	58	9.6

### Vaccination status of the students and reasons for non-vaccination

A majority (86.50%) of respondents were vaccinated against hepatitis B leaving 13.5% non-vaccinated. The coverage was 85.3% among medical, 80.2% among dental and 92.7% among nursing students (BSc. and CN combined). Among those vaccinated, 83.7% (436/521) were fully immunized, i.e. they received 3 or more doses of vaccination, whereas 16.3% (85/521) were only partially immunized. When we asked about the reasons for non-vaccination, 43.2% of the non-vaccinated group stated there was lack of vaccination programs and 21% told that they never thought about it.

### Needle-stick and Sharps-related Injuries (NSSIs)

#### Prevalence of injury

Out of 210 students in clinical rotations, 90 (42.8%) had experienced at least one injury. Among those 90, 63 (70%) were nursing students (B.Sc. and Certificate Nursing), 18 (20%) were medical interns and 9 (10%) were dental interns. In a different revelation, 33 students (15.7%) had also experienced the splashes of patient’s body fluids. Such splashes occurred with patient’s blood in 24 (72.8%), amniotic fluid in 2 (6.0%) and other types of fluid in 7 students (21.2%).

Among those 90 students who had NSSI, two students reported injury or exposure to HIV positive patient (2.2%) and four students to HBV positive patient (4.4%). More than half of the remaining students were unsure about the serology status of source patient.

Likewise, 39 sustained the injury only once, 33 sustained it twice, 12 sustained it thrice and 3 sustained it even four times till the date of survey. Remaining 3 students did not disclose the frequency of injuries. A detail of their first incident was utilized to know the patterns of Needle-stick and Sharps-related injuries in the hospital.

### Type of instrument involved in injury

More than half (57.8%) of those 90 students sustained injury with Hypodermic/Hollow bore needle whereas 27.8% sustained injury with Suturing needle (Table 2).

**Table 2:** **Type of Instrument involved in injury (n = 90)**

Description	Category	Frequency	Percentage
Types of needle/sharps	Hypodermic needle	52	57.8
	Suturing needle	25	27.8
	Scalpel/blade	3	3.3
	IV catheter	3	3.3
	Wire/Force/Retractor	5	5.6
	Dental sharp	2	2.2

### Place and procedure related to injury

A majority (41.1%) of injuries occurred in Internal Medicine followed by Obstetrics-Gynecology & Labor wards (23.3%). The most common procedure that inflicted injury was while drawing patient’s venous blood (31.1%) followed by while conducting delivery in the labor room (17.8%) (Table 3).

**Table 3:** **Details of the first incident of injury (n = 90)**

Description	Category	Frequency	Percentage
Department in which injury occurred	Internal Medicine ward	37	41.1
	Surgery ward	12	13.3
	ObGyn ward and Labor room	21	23.3
	Pediatrics ward	2	2.2
	Emergency Department	2	2.2
	Orthopedics ward	3	3.3
	Dental department	9	10
	Laboratory	4	4.4
Procedure involved during injury	Drawing venous blood for lab	28	31.1
	Opening Intravenous access	11	12.2
	Suturing wounds	4	4.4
	Conducting labor	16	17.8
	Drawing arterial blood (ABG)	3	3.3
	Measuring blood sugar level with Glucometer	4	4.4
	Others	12	13.4
	Dental extraction	8	9.0
	Lab works-Hematology	4	4.4

The most common circumstance leading to injury was while manipulating needle back and forth into the patient for venous or arterial access (35.6%) followed by while administering medication thru intravenous or intramuscular route (24.4%). Likewise, common causes of injury as cited by the respondents were uncooperative patient (33.3%) and stress during work (16.7%). Some 16.7% stated that the injury happened while carrying out the particular procedure for the first time (Table 4).

**Table 4:** **Circumstances and causes of injuries and exposures (n = 90)**

Description	Category	Frequency	Percentage
Most common circumstance leading to injury	While recapping used needle	17	18.9
	While cleaning up the procedure area and disposal of instruments	8	8.9
	While administering	22	24.4
	medications thru IV/IM route		
	While manipulating needle back and forth in the	32	35.6
	patient for venous/arterial		
	Access		
	Collision with needles or	3	3.3
	sharps left in improper places		
	Accidentally injured by others	2	2.2
	While assisting operation	1	1.1
	Other circumstances	5	5.6
Most common cause of injury	Stress at work	15	16.7
	Particular procedure for the first time	15	16.7
	Over confidence for particular procedure	5	5.6
	Not using safety precautions	11	12.2
	Uncooperative patient	30	33.3
	Understaffed situation	4	4.4
	Carelessness/Inattentiveness	4	4.4
	Others	6	6.7

### Tendency of seeking post-exposure prophylaxis (PEP) after an exposure

There was a low tendency of consultation and reporting to the concerned authority after getting exposed to patient’s blood through injury. Less than a half (41.4%) of respondents consulted with their seniors. The tendency of seeking PEP was also found to be very low. Only 10 students (11.1%) revealed that they took PEP either in the form of Tetanus toxoid vaccination or oral antibiotics. Two students who were exposed to HIV positive patients had started taking anti-retroviral prophylaxis. Likewise, only one-fifth of the exposed ones (18 out of 90) went for a post-exposure serological test (HIV, Hep B or HCV). To find out the result of such test and its sequel was beyond the scope of our study.

Chi-square analysis of different variables has been shown in Tables 5 and 6. Hepatitis B vaccination status of the respondents was significantly associated with faculty (p = 0.024) and gender (p = 0.007) (Table 5).

**Table 5:** **Association of Hepatitis B vaccination status with the socio demographic variables (n = 602)**

Characteristics	Category	Vaccination against Hepatitis B		p-value
		Yes	No	
Faculty	MBBS	290 (85.3)	50 (14.7)	
	BDS	77 (80.2)	19 (19.8)	**0.024**
	B.Sc. Nursing	67 (91.8)	6 (8.2)	
	C. Nursing	87 (93.5)	6 (6.5)	
Age Group (years)	≤22 yrs	328 (86.5)	51 (13.5)	0.999
	>22 yrs	193 (86.5)	30 (13.5)	
Sex	Male	276 (83.1)	56 (16.9)	**0.007**
	Female	245 (90.7)	25 (9.3)	
Nationality	Nepalese	472 (86.8)	72 (13.2)	0.628
	Indian	49 (84.5)	9 (15.5)

**Table 6:** **Association of NSSIs with socio demographic variables (n = 210)**

Characteristics	Category	NSSIs		p-value
		Present	Absent	
Faculty	MBBS	18 (64.3)	10 (35.7)	
	BDS	9 (56.3)	7 (43.8)	**0.042**
	B.Sc. Nursing	29 (39.7)	44 (60.3)	
	C. Nursing	34 (36.6)	59 (63.4)	
Age Group (years)	≤22	51 (34.0)	99 (66.0)	**<0.001**
	>22	39 (65.0)	21 (35.)	
Sex	Male	24 (61.5)	15 (38.5)	
	Female	66 (38.6)	105 (61.4)	**<0.001**
Nationality	Nepalese	87 (42.4)	118 (57.6)	0.653
	Indian	3 (60.0)	2 (40.0)	

Needle stick and sharps-related injury was significantly associated with faculty (p = 0.042), age (p < 0.001) and gender (p < 0.001) (Table 6).

## Discussion

Hepatitis B vaccination coverage among medical school students in our study was 86.50% which is comparable to a study carried out in Malaysia (85.5%) [7]. The vaccination rates in two different medical schools of Pakistan were 57% and 42% respectively [8, 9]. A previous study of similar kind but conducted among Health care workers in Nepal found the vaccination rate to be 60% [10]. In this light, our finding somehow indicates the rising awareness on occupational health, hazards and preventive measures.

In our study, a majority of the students from each faculty (medical, dental and nursing) were found have vaccination against Hepatitis B. Almost all students had had this privilege in Canada [11]. In contrast, surprisingly only 1.9% of nursing students had had vaccination in a medical school of Orissa, India [12].

Lack of effective vaccination programs in our country was the main reason cited for non-vaccination (43.2%). In Pakistan, the reason was high cost of vaccination (44.7%) and a false belief among students that they were not even at risk (33.7%) [9]. Asif M et al., in another study, reported that students did not receive vaccination because there was a lack of motivation among peers (29.2%) and some even did not feel the need of it (24.8%) [8].

The prevalence of NSSI among trainee students in our institute was 42.8%. A previous study carried out in Nepal but among health care workers reported it to be 74% [10]. However, it cannot be stated that the prevalence has really reduced. Our study targeted the trainee students who might have failed to recall and report the injuries or simply hesitated to reveal it for personal reasons.

Our finding is in accord with that in Mongolia where the NSSI prevalence was 38.4%. However, it is higher when compared to teaching hospitals in India (32.75%) and Nigeria (31.25%) [13–15].

A Canadian study reported that the highest rate of injury was among dental students (82%) followed by medical (57%) and nursing students (27%) [11]. In our study, however, the nursing students (70%) had the highest rate of injury followed by medical interns (20%) and dental interns (10%). Since interns are mostly engaged in hands-on training, the injury rate could be higher in reality. This low rate, however, could be attributed to their low response rate. More than a half of them could not return questionnaires on time because they had busy schedules rotating in the teaching district hospitals at periphery.

High exposure rates to HIV (2.2%) and HBV (4.4%) positive cases in our study clearly highlight the vulnerability of health care workers including clinical trainees in Nepal. According to World Health Organization (WHO), annual proportion of health-care workers who get exposed to blood borne pathogens is 0.5% for HIV, 5.9% for HBV and 2.6% for HCV [6].

Regarding NSSIs, a majority of incidents occurred with Hollow bore needles. Manipulation of needle back and forth into an uncooperative patient was the main circumstance of injury. Our findings are supported by two studies, one done in Australia and the other done by Centers for Disease Control and Prevention in USA [16, 17]. Trainees in our study usually had incidents in Internal Medicine ward as in Malaysia [7]. This could be because drawing venous blood sample from patient is the first procedure students get to learn in wards. Opening IV lines and suturing cut wounds are among other procedures which students seem to struggle with.

Although recapping the used needle has been discouraged and even prohibited by National Institute of Occupational Safety and Health (NIOSH, CDC) since 90’s, around 3% of NSSIs in US are still due to this practice [17]. Recapping led to 19% of injuries in our study. For many years, it has been a topic of debate especially in low income and technically poor countries like Nepal. Some health professionals argue that recapping may prevent the unexpected injuries both to the handler and to the people around him. Such myths need to be resolved soon and carrying out similar studies in a large scale might be a good initial step.

Regarding Post-exposure prophylaxis (PEP), only 11.1% of those exposed took it either in the form of Tetanus toxoid or oral antibiotics. This lower rate could be attributed to low reporting tendency among students. A survey done by Perry J and Robinson S.R among nurses found that 15% of exposed took PEP [18].

In our study, Hepatitis B vaccination status of the respondents was significantly associated with socio-demographic variables like faculty and gender and NSSI incident was significantly associated with faculty, age and gender. These findings are in accord with studies carried out by Shokier et.al in Kuwait [19].

The present study had some limitations. Though each respondent was assured of confidentiality, there was still a chance of bias in reporting, as the data were collected using self-administered questionnaires. Recall bias was another possibility. Some students might not have remembered the exact number of immunization doses they took during childhood. Regarding NSSIs, some trainees probably overlooked minor injuries while others reported about incidents that did not involve patient’s blood. This study did not include all HCWs in the hospital (viz. Hospital assistants, Staff nurses, Residents and Staff physicians). The temporal association between variables could not be established as the study design was cross-sectional.

Nevertheless, several positive aspects of this study; one being it strength in raising occupational awareness in health institutions, could fairly overcome the limitations. The particular study could also be utilized as the resource first to carry out in-depth studies and then to formulate new health policies and regulations in Nepal.

## Conclusion

The study revealed that a majority of students in the institute were vaccinated against Hepatitis B. However, as a matter of concern, those who were not vaccinated could be at higher risks of acquiring infections if they inflicted injuries with infected patients. Therefore, the health policy makers and executives must immediately address the common reasons that have been cited for non-vaccination.

Another alarming situation that needs to be tackled is high rate of NSSI among trainees, accompanied by a low tendency of Post-exposure prophylaxis and serology tests. A considerably high HIV and HBV exposure rates in the study calls for an extensive educational reinforcement on Universal precaution.

### Ethical clearance

Institution Ethical Review Board, BP. Koirala Institute of Health Sciences, Dharan, Nepal.

## Authors’ information

SB is a Medical graduate from BPKIHS. He carried out this study during his Internship in 2012. He worked as a Medical Officer in Nepal for a couple of years and currently holds a position of Visiting Scholar in Cleveland Clinic Foundation, Ohio. He has interests in Infectious diseases, Injuries and Global health. SKC has been working as Program Associate in HIV & SRHR Coalition Nepal and conducts trainings on HIV and STIs to raise awareness among vulnerable groups. PMSP is Consultant Senior Public Health Officer in Influenza Surveillance Project at Patan Academy of Health Sciences (PAHS), Nepal. He is also a Lecturer at the Department of Community Health Sciences at PAHS. He received his Post-graduation degree in Community Medicine and Tropical Diseases from BPKIHS. SL is Professor and Head of Nursing education in BPKIHS. SR is Professor in the Department of Internal Medicine, BPKIHS and the Chief of Tropical and Infectious Disease Division. He is better known as the Scientist for his great contribution in the field of Infectious diseases in Nepal.
